# Building Capacity for Evidence-Informed Priority Setting in the Indian Health System: An International Collaborative Experience^☆^

**DOI:** 10.1016/j.hpopen.2020.100004

**Published:** 2020-03-13

**Authors:** L.E. Downey, S. Dabak, J. Eames, Y. Teerawattananon, M. De Francesco, S. Prinja, L. Guinness, B. Bhargava, K. Rajsekar, M. Asaria, N.V. Rao, V. Selvaraju, A. Mehndiratta, A. Culyer, K. Chalkidou, F.A. Cluzeau

**Affiliations:** a Global Health and Development, School of Public Health, Imperial College London, London, United Kingdom; b Health Intervention Technology Assessment Program (HITAP), Bangkok, Thailand; c School of Public Health, Post Graduate Medical Institute of Health Education and Research (PGIMER) Chandigarh, India; d Department of Health Research, Ministry of Health and Family Welfare, Government of India, New Delhi, India; e London School of Economics and Political Science, London, United Kingdom; f Centre for Health Economics, University of York, York, United Kingdom; g Centre for Global Development Europe, London, United Kingdom

**Keywords:** Capacity Building, Health Economics, Health Technology Assessment, Health policy, India

## Abstract

India’s rapid economic growth has been accompanied by slower improvements in population health. Given the need to reconcile the ambitious goal of achieving Universal Coverage with limited resources, a robust priority-setting mechanism is required to ensure that the right trade-offs are made and the impact on health is maximised. Health Technology Assessment (HTA) is endorsed by the World Health Assembly as the gold standard approach to synthesizing evidence systematically for evidence-informed priority setting (EIPS). India is formally committed to institutionalising HTA as an integral component of the EIPS process. The effective conduct and uptake of HTA depends on a well-functioning ecosystem of stakeholders adept at commissioning and generating policy-relevant HTA research, developing and utilising rigorous technical, transparent, and inclusive methods and processes, and a strong multisectoral and transnational appetite for the use of evidence to inform policy. These all require myriad complex and complementary capacities to be built at each level of the health system . In this paper we describe how a framework for targeted and locally-tailored capacity building for EIPS, and specifically HTA, was collaboratively developed and implemented by an international network of priority-setting expertise, and the Government of India.

## Highlights

• UHC requires explicit trade-offs to maximize health using limited available resources
• Health Technology Assessment (HTA) is the gold-standard for informing such trade-offs
• There is a dearth of skills required to conduct and utilize HTA evidence in India
• We describe the first National capacity-building program for HTA in India
• The approach described presents a strong model for adaptation in similar contexts

## 1 Background

India has approximately one sixth of world population and has experienced marked economic growth over the last decade [1]. This rapid economic growth has been accompanied by slower improvements in population health, with a growing burden of chronic non-communicable diseases, and high rates of child malnutrition, stunting and maternal and neonatal mortality across many States [2., 3., 4.]. As India’s burden of chronic conditions continues to rise, so too does the cost of care [5., 6., 7., 8.] yet, the healthcare budget, at 1.3% of GDP, is one of the lowest in the world [9]. Given the need to reconcile the achievement of Universal Coverage (UHC) and the Sustainable Development Goals [2,4,10] with a realistic appreciation of the resources that are likely to be available, a robust mechanism is required through which to set priorities for health investment based on strong evidence to maximise the impact on population health.

Health Technology Assessment (HTA) is the internationally-recognised gold standard for systematically synthesizing evidence of the clinical and cost-effectiveness of health interventions [11]. Systems for utilising HTA to improve allocative efficiency are increasingly being established in Asia, South America, and more recently, Africa ( [12., 13., 14., 15., 16., 17., 18.]. HTA is credited as a key contributor to Thailand’s successful Universal Health Coverage (UHC) program, where it is a central component of decision-making for including and excluding benefits in the country’s UHC Scheme and its National List of Essential Medicines [7,9,10]. The World Health Organisation signed a resolution in 2014 supporting the importance of HTA as a component of achieving Universal Health Coverage [4,11], thereby elevating the global recognition of HTA as a lever for health system reform.

India has formally committed to institutionalising HTA as an integral component of the priority setting process in the 12th Five Year Plan [12], the National Institute for Transforming India (Niti Aayog) 3 year Action Plan [19], and the National Health Policy [20]. This political commitment to HTA comes at an opportune time, as the government rolls out the country’s National Insurance Scheme, Ayushman Bharat Yojana or Pradhan Mantri Jan Arogya Yojana (AB-PMJAY), which covers some 1500 packages of secondary and tertiary medical care for 500 million beneficiaries [21,22]. The government of India, via the Department of Health Research (DHR), has established a national HTA body, HTAIn, to coordinate the HTA function within the Ministry of Health and Family Welfare [23,24] and institutionalise HTA as part of the evidence-informed priority setting process for health policy decisions.

A robust HTA mechanism requires a skilled cadre of local professionals adept at commissioning and generating policy-relevant HTA research, developing and utilising rigorous technical, transparent, and inclusive methods and processes, and a strong multisectoral and transnational stakeholder appetite for the use of evidence to inform policy [16,26] [27., 28., 29., 30.]. Given the recent establishment of the HTAIn and nascent introduction of HTA into the Indian health system, there is a presumed dearth of HTA-specific local skills and expertise. For example, local technical professionals play an essential role in producing HTA evidence, however there is a known marked absence of health economists with expertise in HTA in India [25]. This poses a significant challenge to the successful generation and deployment of HTA evidence in the country [4,18].

The Government of India approached the International Decision Support Initiative (iDSI), a global network of health, policy and economic expertise [26], to support a National program for targeted capacity building for HTA and evidence-informed priority-setting. This paper describes the locally-tailored capacity building approach for HTA in India over a period of four years (June 2015 – June 2019), drawing on a recognized capacity building framework and bringing together different elements of the capacity building process for strengthening the system of evidence-informed priorty setting in India.

## 2 Methods

### 2.1 Framework for capacity building

The capacity building framework is described previously by Li and colleagues [26] and embodies five iterative steps: i) engaging stakeholders in capacity development; ii) assessing capacity needs and assets; iii) formulating a capacity development response; iv) implementing a capacity development response; v) evaluating capacity development [26].

#### 2.1.1 Engaging Stakeholders

A stakeholder mapping exercise was initially undertaken in partnership with the Indian Council of Medical Research (ICMR) and the DHR. A comprehensive list of HTA stakeholders was drafted that spanned government and non-government institutions, multilateral development organisations, donors, industry representatives, clinical care representatives and civil society advocates. These stakeholders were invited to attend a large stakeholder consultative workshop held in Delhi in July 2016 which was jointly convened by DHR, ICMR, and iDSI. A list of attendees is available online [27]. The workshop was attended by over 200 delegates, including both Secretaries of State and a number of State-level Health Secretaries. This provided an opportunity for stakeholders to share experiences and discuss the context, need, function, structure, and future plans for embedding HTA in Indian health policy [28].

#### 2.1.2 Assessing Capacity Needs and Assets

##### 2.1.2.1 Technical Capacity: Evidence generation

To assess the competence of technical skills in India skills and inform strategic planning for teaching and training, it was necessary to i) identify institutions with existing capacity in HTA and related disciplines; and ii) identify gaps in existing technical capacity to undertake HTA. We developed a structured questionnaire which was distributed by the DHR [29] to sixty publicly-funded institutes with a role in health education and research, identified in the aforementioned stakeholder mapping exercise and opportunistically through discussions held during the stakeholder workshop. The questionnaire asked institutes to give information on formal staff qualifications and applied experience in HTA and related fields, such as health economics, statistics, epidemiology, pharmacology, policy analysis and systematic review. Institutes were also requested to submit any relevant publications.

In addition, we made a formal assessment of the availability and completeness of the evidence necessary to inform the conduct of HTA in India [30]. Local data requirements were outlined according to six domains (clinical effectiveness, cost, service use, equity, clinical epidemiology and quality of life).

#### 2.1.3 Formulating a Capacity Development Response

The information gathered during the capacity needs and assets assessment was used to develop a comprehensive capacity building program, which was agreed between iDSI and the DHR in 2016 [31]. Capacity requirements were then monitored for change throughout the program and adapted when required, in consultation with DHR.

Forty two (70%) institutes responded to the technical capacity questionnaire . Responses were analysed and scored by DHR according to pre-defined criteria [32]. Research related to HTA submitted by respondents was collated and synthesized by DHR to form the basis of a publicly-accessible National HTA study repository [33]. Key findings indicated a high level of interest in engaging with the Government of India in HTA skill development. Many institutes indicated that staff had strong HTA-related skills in epidemiology, statistical analysis and systematic review, however almost all indicated that staff would require further training in economic analysis and, in particular, in cost-effectiveness analysis.

The data gap analyses revealed that there was a dearth of data necessary for the local conduct of HTA, most notably for healthcare costs, and detailed information on this is published elsewhere [30].

The capacity development response was designed to target the specific needs within each level of the health policy ecosystem: the individual, the node, the network, and the environment (INNE) [34]. The strategy was modified in 2017 and 2018 to accommodate feedback from the DHR and local partners in response to changing local capacity building requirements.

#### 2.1.4 Implementing a Capacity Development Response

Table 1 describes each of the key capacity building activities delivered by the iDSI over the intervention period.

**Table 1:** Targeted capacity-building activities delivered and/or facilitated by iDSI

Environment	Network	Node	Individual
Convened and facilitated participation of Senior Indian dignitaries in numerous high-level HTA-relevant meetings:	Supported convening of HTA technical partner network (TPN)	Participated in UK-India joint steering committee for HTA	Delivered series of training workshops for the HTAIn and TPN
- PMAC (2016, 2017, & 2019)	Co-facilitated major HTA conference in India	Participated in HTA advisory group meetings	Continuous technical assistance to HTAIn and TPN towards their HTA work
- NICE & HITAP study visits(2015 – 2016)	Introduced HTAin to 2 major HTA networks:	Long-term staff presence to provide consistent advice and support	Funded 2 x MsC Scholarships for Indian students
- iDSI South-South workshop (2017)	- Health Technology Assessment international (HTAi)	Document sharing and feedback/quality appraisal	Internship opportunities at HITAP Thailand
- ICMR/DHR/iDSI stakeholder workshop (2016)	- HTAsialink	Technical assistance towards key process and procedural developments	PhD studentship co-supervised by HITAP Thailand
		Supported program of HTA research and development of National cost database	

##### 2.1.4.1 Environment

Environmental capacity development focused on facilitating recognition of the political economy of HTA and its important role in shaping the health policy agenda towards setting more transparent, inclusive, equitable, and evidence-based priorities for health spending and care delivery. This kind of awareness raising was facilitated by way of delivery of a series of high-level international knowledge-exchange forums, hosting study-tours for Indian officials, and through maintaining a regular dialogue between capacity building partners and senior Indian policymakers through a UK-India Joint Steering Committee for HTA.

Senior policymakers from the Indian Ministry of Health and Family Welfare and DHR were supported to attend high-level knowledge exchange events such as the Prince Mahidol Awards Conference in Bangkok 2016 [35] and an iDSI South-South Workshop in Johannesburg in 2017 [36]. These events introduced attendees to a range of nascent, developing, and developed country HTA systems. In addition, in 2015 [37] and 2017 [38], study tours were arranged for a DHR-led group to travel to two world-renowned HTA institutions: The National Institute of Health and Care Excellence (NICE) UK, and the Health Interventional Technology Assessment Program (HITAP) Thailand.

##### 2.1.4.2 Network

The capacity gap analysis enabled the DHR to identify a network of technical research partners across the country. To date, six Regional Resource Centres have been established by DHR in collaboration with the State Governments across the country [39]. These centres are fully funded by the DHR to undertake HTA for the HTAIn as part of a Technical Partner Network (TPN). The technical lead of each Resource Centre liaises directly with their respective State Governments to sensitize them to the role of HTA in priority setting and inform the topics submitted by the State to the HTAIn for formal assessment [39,40].

Network-level capacity building activities focused on facilitating linkage between HTA practitioners at the national level and internationally via Indian participation in global HTA forums. The Post Graduate Institute of Medical Education and Research Chandigarh (PGIMER) convened a large National HTA conferences in India in collaboration with DHR, iDSI and the National Health Systems Resource Centre [41]. This forum was attended by over 100 participants and brought together the local and extended networks of HTA practitioners. Participation by HTAIn was facilitated in two major international forums: HTAinternational Conference, and HTAsialink. In the former, a panel was convened which included two members of the DHR, one from PGIMER Chandigarh, and one from iDSI, to discuss the establishment of HTAIn and outline plans for building capacity [42]. At HTAsialink, two members of the HTAIn technical partner network were invited to present their HTA research in 2017 [44] and 2018 [45], which provided exposure to a community of HTA researchers across Asia and the opportunity to receive real-time feedback on their work from this international HTA community.

##### 2.1.4.3 HTA Node at National level

Technical assistance was provided to the DHR for establishing the HTAIn as a functional core agency dedicated to coordinating a National HTA function for India [43]. This included providing a sounding board for senior staff to share ideas and seek advice, sharing key documents and publications for local adaptation, creating opportunities for knowledge exchange with others in a similar stage of their HTA journey in neighbouring countries, and learning from participation in international HTA forums ( [38,44., 45., 46.]). Direct support was also given to the HTAIn and PGIMER Chandigarh in the development of methods and process documents to guide uniform conduct of HTA by all technical partners. These documents included a Reference Case and associated Methods Manual, an HTA guide book, and operating procedures for managing conflicts of interest and stakeholder consultation (see https://dhr.gov.in/HTAIn-documents).

Technical assistance was provided by experts (international and local) permanently based in Delhi, who met with DHR frequently. A wider pool of senior international experts visited India on a semi-regular basis.

The data gap analysis informed a research capacity building program and a collaborative program of work between the iDSI and the PGIMER Chandigarh on a national cost database. This will provide a nationally relevant repository of primary cost information for HTA practitioners and the wider health policy and research community [47].

##### 2.1.4.4 Individual

Individual capacity building interventions were targeted at building a skilled cadre of indigenous health research practitioners. Training was delivered through a series of technical skill-building workshops, supplemented by intensive analytical support. Skills building workshops consisted of four comprehensive training modules (introduction to HTA; systematic review and evidence synthesis; decision analytic modelling; sensitivity analysis and communicating results). Key topics include, but are not limited to: The role of HTA in priority setting for UHC; Introduction to cost effectiveness analysis; the Quality Adjusted Life Year (QALY) and disability adjusted life year (DALY); Health care costing; Designing a study scope; Designing a search strategy; Identification and quality appraisal of evidence; Meta analyses; Decision tree models; Markov models; Designing a complex markov model; Populating and running a markov model; Interpreting model outputs; Probabilistic and one-way sensitivity analyses; Writing-up results for publication; and drafting policy briefs. Training courses ranged from 4 to 6 days each, designed and delivered over 18 months, with support from PGIMER Chandigarh, the HTAIN, and the Indian Council of Medical Research [48,49]. These modules were delivered to roughly 30 HTAIn technical partners. The partners were all funded by the HTAIN to undertake policy-relevant HTA research.

Two scientists from 2 separate Technical Partner institutes were selected by the Director General of the Indian Council of Medical Research for IDSI scholarships to study a 2-year MsC program in Health Technology Assessment at Mahidol University. The students have tied placements within their respective institute and are expected to contribute towards training their peers. The Health Intervention Technical Assessment Program (HITAP) of Thailand co-supervise one Indian PhD student with a thesis focused on HTA and EIPS, and facilitated a 6 month internship for an Indian MsC student for 6 months. Both the PhD and MsC students are affiliated with PGIMER Chandigarh and the HTAIn Technical Partner Network.

#### 2.1.5 Evaluating Capacity Development

**Fig. 1:**
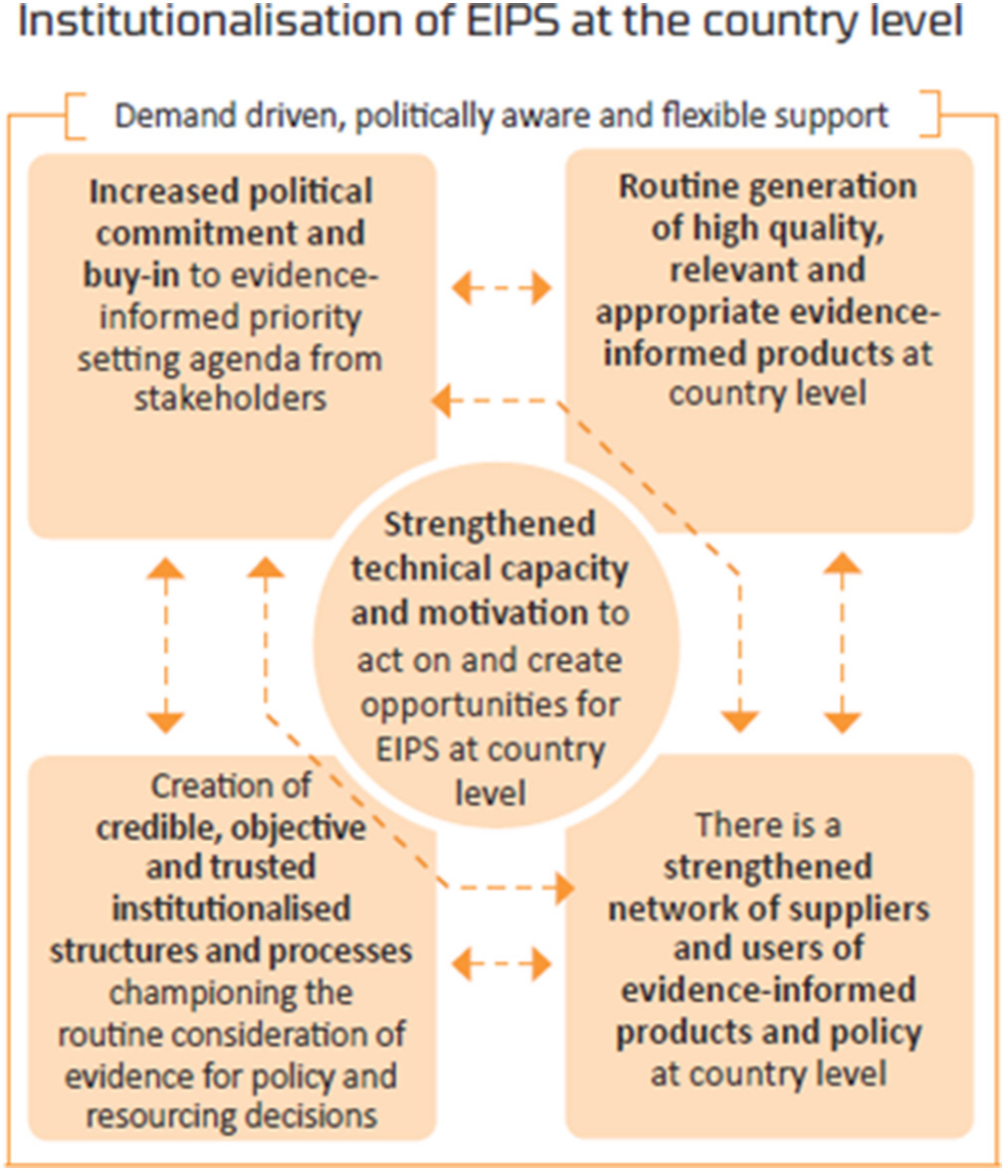
iDSI Theory of Change: Institutionalisation of Evidence-Informed Priority Setting at the Country Level

A comprehensive monitoring evaluation and learning (MEL) framework was used to guide evaluation of the effectiveness of these capacity building activities [50]. The MEL framework captures the design, structure, and execution of the capacity building program from a broad organisational framework, represented schematically in Fig. 1.

#### 2.1.6 Measuring impact

Table 2 provides a summary of the key milestones in the development of HTA in India over the course of the study period.

**Table 2:** Key HTA milestones 2016 - 2019

Environment	Network	Node	Individual
HTA officially recognised in all key policy documents: National health Policy (2018); ICMR strategy (2017 – 2021; Niti Aayog Vision (2017)	Technical partner network (TPN) established and fully operational	Core HTAIn team has been established and continues to grow	HTAIn staff contribute to training of TPN peers
5 HTA studies have been completed and ratified by government	First national HTA conference held and well-attended (2018)	Appraisal committee operational and convened regularly	Approximately 30 TPN staff have completed all 4 training modules in HTA
2 Completed HTA studies have directly informed policy decisions to date	HTAIn participation in 2 major international HTA fora: HTAinternational and HTAsialink	Uniform methods and procedures developed and routinely being put into practice	

## 3 Pillar 1: Strengthened technical capacity and motivation to act on and create opportunities for evidence informed priority setting

HTAIn established and maintained an active network of technical collaborators throughout India. A comparative assessment of pre and post training feedback indicates that while almost all individuals started with zero knowledge of systematic reviewing for HTA, a high proportion of respondents indicated that they had gained important knowledge and put this into practice. Members of the HTAIn team themselves started with zero exposure to HTA but by the third training event were contributing as co-facilitators by teaching concepts of HTA to their peers.

## 4 Pillar 2: Increased political commitment and buy-in to evidence informed priority setting agenda from stakeholders

There has been increasing political recognition of HTA as an important tool for UHC in India. Prior to the formal collaborative partnership between iDSI and the government of India, HTA was mentioned only in the draft of the National Health Policy document. HTA is now recognised as an integral component of the Government’s health strategy, with explicit reference to HTA in all key national health policy documentation, including the National Health Policy (2017 [20]) , the Niti Aayog forward view (2017 [19]) and the Indian Council of Medical Research strategic vision (2017 [51]). Senior representatives of both the National and State Governments of India participated as active stakeholders throughout the duration of this engagement, attending high-level awareness raising events, technical training workshops, stakeholder consultation meetings, and have communicated with the media regarding the importance of HTA in PMJAY [52,53].

## 5 Pillar 3: Routine generation of high quality, relevant, and appropriate evidence informed products

Results from the first two HTA studies were swiftly adopted into policy, with the intraocular lens replacement for cataracts analysis [54] informing the ophthalmology package of services under the PMJAY; and the government of Andhra Pradesh electing to invest in auto disposable syringes [55,56]). A new health research environment has also emerged, where the HTAIn, under the DHR and with the assistance of iDSI and PGIMER Chandigarh, has been able to identify gaps in available evidence for the effective conduct of HTA [30] and commission comprehensive research to address one of those gaps related to cost information by supporting the development of a National Cost database and facilitating a Nation-wide costing study, coordinated by the DHR [47].

## 6 Pillar 4: Creation of credible, objective and trusted institutionalized structures and processes championing the routine consideration of evidence for policy and resourcing decisions

The HTAIn is now fully functional [43] [23] and the HTAIn team has expanded from a single staff member to a team of more than a dozen within a short space of time. Important documentation has been developed, adhered to, and updated within this short timeframe, including standard operating procedures, a process guide, a methods manual, a reference case for economic evaluation, a conflict of interest policy, and a guideline for stakeholder consultation ((see https://dhr.gov.in/HTAIn-documents). The HTAIn actively engages in raising awareness of the team and its function within the health policy and academic community, commissioning HTA studies, setting key standards and processes, teaching and training local researchers, facilitating quality assurance of HTA through a technical appraisal committee, and championing evidence-informed health policy.

## 7 Pillar 5: Strengthened network of suppliers and users of evidence informed evidence informed products and policy

A network of HTA suppliers has been established and strengthened. HTAIn has signed MoUs with at least eight State governments [39] and State-level technical partners for HTA exchange. HTAin commissions a technical partner to undertake a HTA of a given topic, the technical partner conducts the HTA under HTAIn supervision and support, and results of the final HTA study are then made publicly available once approved by the HTAIn appraisal committee and Ministry of Health and Family Welfare [39,40]. While such a process is still being tested and finetuned, all completed and commissioned HTAs to date have been demand-driven by policymakers, and the HTAIn have continued to receive HTA topic requests from both State and Central government partners (see https://dhr.gov.in/HTAIn-documents).

## 8 Discussion

As India progresses towards its goal of Universalising health coverage, continuing high level political commitment to HTA will be important to ensure the streamlining of evidence into policy priorities. The decentralised health system, geographical and cultural diversity, and sheer population size of the country coalesce to create a unqiely challenging environment for establishing and maintaining systemic reform. The central placement of the HTAIn within the Ministry of Health and Family welfare gives credibility to HTA as a meaningful mechanism to informing National Policy. Moreover, the collective engagement across a network of partners also creates a strong foundation for linking the National initiative to the States through the establishment of Regional Reseource Centres across the country.

From a technical perspective, the ability to equip a network of institutions and individuals to acquire competence in HTA in a country as large and diverse as India is necessarily limited by way of time and human resource constraints. International best practice for education in this specialism is a 1-2 year full-time Masters degree, followed by at least two years professional experience in the field. Every effort was taken throughout the capacity building program to deliver high-quality hands-on training and practical skill building to the HTAIn and its network. The skills learned during these training workshops have been put into practice by attendees, the majority of whom have contributed towards undertaking a HTA from start to finish. This is impressive considering the uniform null experience in HTA at the inception of the capacity building program.

An important challenge for the successful deployment of HTA into the Indian political system is the myriad users for this kind of evidence within the highly fragmented Indian health system [23,40]. Long-term success will depend on how effectively HTA is able to cater to the different types of decision-making within this complex system. Exposure to international policy and practitioner HTA communities provides an important opportunity to recognise how other countries have utilised HTA evidence, and where HTA can best be leveraged to strengthen the Indian health system. This kind of global knowledge exchange, facilitates peer-to-peer learning in a way that both inspires ideas for local appropriation, but also creates opportunities to understand what has not worked for countries in their HTA journey and why, so that the same pitfalls can be avoided.

We identify three key factors contributing to the achievements made throughout the rollout of the capacity building program: strong political will and domestic financial commitment; detailed baseline capacity assessment and data collection; and comprehensive and flexible engagement between all collaborative partners and beneficiaries throughout the period of study.

## 9 Practical and political commitment to HTA

Throughout the intervention period, the DHR made significant human and financial resources available for the HTAIN capacity building program. This commitment allowed the collaborative capacity building program to flourish at a practical level by facilitating the hiring of dedicated HTA teams across the country and covering the costs incurred of holding multiple training events.

## 10 Evidence-informed capacity building approach and local context

The importance of establishing a baseline and taking the time to understand local practices and existing or potential future bottlenecks is often overlooked. However, the time invested in capacity assessment and strategic planning ensured HTA capacity building was targeted towards local needs and requests, which ultimately improved uptake and buy-in at each level of the HTA ecosystem.

## 11 Collaborative and flexible engagement between all parties

The successful implementation of the capacity building program was made possible through the full engagement of partners. Incentives were important contributors to partners continued engagement. Technical partners and the beneficiaries of the technical training had salaries paid by the DHR as Regional Resource Centre employees within a HTA technical Partner Network, with clear terms to engage in work solely related to HTA. Moreover, given the political interest in HTA, engagement with the HTAIN offered technical partners unparalleled closeness to policymakers and a direct link between policy needs and research. The sustainability of HTA in India will depend on technical supply meeting what is expected to be an increasing demand for evidence-informed value for money in public insurance packages, including the PM-JAY [57]. This will require continual local development of a cadre of skilled multidisplinary professionals.

## 12 Conclusions

The locally-tailored and collaborative capacity building approach described here presents a model for targeted capacity building in HTA that could be adapted for other low and middle income countries. Successful and sustainable implementation of HTA requires long-term commitment to building both supply and demand-side capacity for the generation and utilisation of HTA evidence.
